# A Complex Case of Infective Endocarditis With Systemic Embolization: Multidisciplinary Approach to Diagnosis and Management

**DOI:** 10.7759/cureus.48461

**Published:** 2023-11-07

**Authors:** Mafalda Vasconcelos, Isabel Cardoso, Alberto Fior, Ana Paiva Nunes

**Affiliations:** 1 Internal Medicine, Hospital Beatriz Ângelo, Loures, PRT; 2 Cardiology, Centro Hospitalar Universitário de Lisboa Central, Lisbon, PRT; 3 Stroke Unit, Centro Hospitalar Universitário de Lisboa Central, Lisbon, PRT

**Keywords:** splenic infarction, mitral valve pseudoaneurysm, thrombolysis complications, ischemic stroke, septic embolism, infective endocarditis

## Abstract

A 44-year-old man with no known medical history presented with stroke symptoms and was found to have occlusion of the M1 segment of the right middle cerebral artery. Thrombolysis and aspiration thrombectomy were successfully performed. However, in the following hours, he developed a fever and multiple cerebral hemorrhages. Due to a drop in hemoglobin post-angiography, an abdominopelvic CT was performed, revealing extensive splenic and renal infarctions. The patient was diagnosed with infective endocarditis (IE) with mitral and aortic vegetations and severe aortic regurgitation. Treatment for IE was initiated, and valve surgery was scheduled after six weeks of antibiotic therapy. Transesophageal echocardiogram documented pseudoaneurysm of the anterior mitral valve leaflet with a high risk of rupture, leading to the decision for early surgery. A prior splenectomy was performed due to the risk of splenic bleeding during anticoagulation for cardiac surgery, being complicated by hemorrhagic shock. The patient ultimately died from complications, including ventilator-associated pneumonia, septic shock, and refractory respiratory failure. Stroke can be the initial manifestation of IE, and the optimal medical and surgical approach must consider the risks of systemic embolization and surgical complications.

## Introduction

Infective endocarditis (IE) is defined as an infection of the endocardial surface or intracardiac devices and is associated with multiple systemic complications. Systemic embolization occurs in 20-50% of IE patients [1], with approximately 25-40% of IE patients experiencing embolic events in the central nervous system (CNS) [2]. CNS embolization can manifest as the initial presentation of the disease and often involves extensive, multifocal lesions with hemorrhagic transformation, leading to severe and debilitating consequences. Prior embolization to the CNS is a factor that influences the decision for surgical intervention. Arterial embolization to other organs and systems can have serious implications on prognosis. It is crucial to highlight the significance of splenic embolization, which can result in infarctions complicated by abscesses or organ rupture, along with complications that affect arterial walls. These complications can potentially lead to the formation of intracerebral or extracerebral mycotic aneurysms.

## Case presentation

We present the case of a 44-year-old man who collapsed in a public space and showed altered speech and weakness on the left side of his body. The prehospital Stroke Code was activated, and he was promptly admitted to the hospital within 40 minutes of the initial observation of neurological deficits. He was originally from Nepal and had lived in an urban area of Portugal for the previous three months. He worked as a restaurant cook and had no known medical history, allergies, or chronic medication.

**Figure 1:**
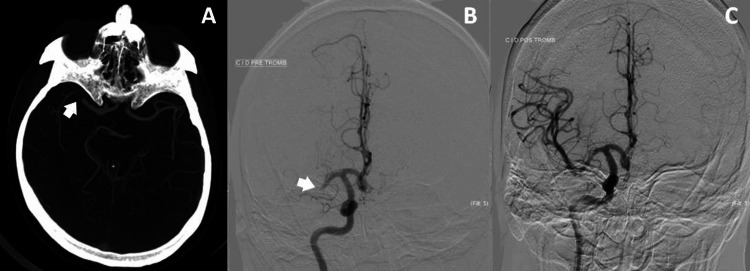
(A) Cranial angio-CT and (B) pre-thrombectomy cerebral angiography documenting an occlusion of the M1 segment of the right middle cerebral artery (arrows); (C) post-thrombectomy cerebral angiography showing reperfusion of the occluded artery

Upon admission to the hospital, the patient presented with a rightward conjugate gaze deviation beyond the midline. Despite demonstrating the ability to identify objects and follow simple commands, the patient exhibited anosognosia, left homonymous hemianopia upon threat reflex, left central facial paresis, left hemiplegia, left hemihyposthesia, and dysarthria with unintelligible speech. The patient achieved a score of 16 points on the National Institute of Health Stroke Scale (NIHSS), a comprehensive assessment tool used to evaluate stroke severity. Furthermore, the patient’s blood pressure, blood glucose levels, and international normalized ratio (INR) were all within normal ranges. After undergoing a cranioencephalic computed tomography (CT) scan (Figure 1A) and subsequent CT angiography (Figure 1B), it was determined that the M1 segment of the right middle cerebral artery was occluded, without any visible acute ischemic changes. He received recombinant tissue plasminogen activator (rtPA) thrombolysis and successful aspiration thrombectomy. Subsequently, complete reperfusion (thrombolysis in cerebral infarction (TICI) scale: grade 3) was achieved without any complications or adverse events. A post-procedure angiography (Figure 1C) confirmed the successful removal of the occlusion, without immediate complications or adverse events.

After following the prescribed procedure, the patient was admitted to the Stroke Unit, where a marked improvement in neurological deficits was observed immediately after angiography. The patient was found to be awake, exhibiting left-sided hemispatial neglect, following simple commands, and having unrestricted eye movement. He showed minor left central facial paresis and performed arm extension without pronation or lower deviation. Moreover, the patient displayed left hemihyposthesia and intelligible dysarthria. The NIHSS score was 6, indicating a moderate stroke.

**Figure 2:**
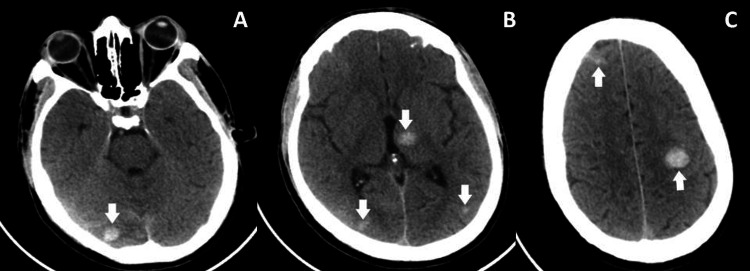
Axial cuts of cranial CT (in the caudocranial direction: A-C) revealing multiple foci of hemorrhagic transformation (arrows)

A few hours later, he developed a fever and altered consciousness. He was only arousable to painful stimuli and unable to produce speech or consistently follow commands. A repeat cranioencephalic CT scan was conducted, revealing hemorrhagic transformation with multiple small corticosubcortical parenchymal hemorrhages in the precentral, mid-frontal, and left temporo-occipital regions, as well as in the posterior parietal and parasagittal occipital regions on the right. Additionally, a small left thalamic hematoma and subtle subarachnoid hemorrhagic components were observed (Figures 2A-2C). Reversal of rtPA was performed using tranexamic acid and fibrinogen according to the institutional protocol.

**Figure 3:**
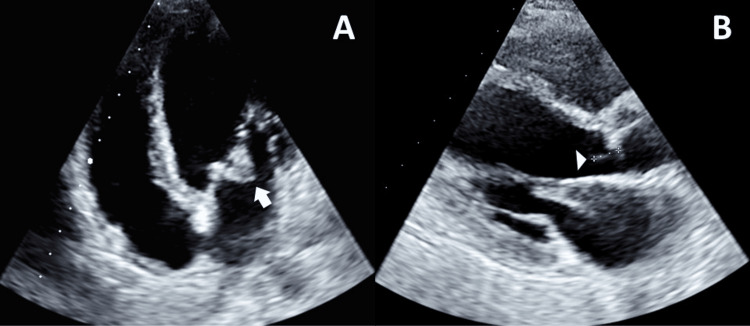
Transthoracic echocardiogram: (A) apical view showing a large vegetation on the mitral valve (arrow) and (B) parasternal long axis view showing a linear vegetation on the aortic valve (arrowhead)

Due to the presence of right lumbar percussion tenderness, persistent tachycardia, and a notable decrease of 2 g/dL in serum hemoglobin levels, medical professionals conducted an abdominopelvic CT scan. This scan revealed the presence of splenomegaly accompanied by multiple areas of splenic infarctions, as well as the diffuse infiltration of hematic material within the right rectus abdominal and oblique muscles. Given the clinical suspicion pointing toward a potential cardioembolic source, a thorough transthoracic echocardiogram was meticulously performed. This echocardiogram uncovered a sessile structure measuring 18 × 12 mm situated on the atrial side of the anterior leaflet of the mitral valve (as shown in Figure 3A). This anomalous structure was found to be responsible for inducing mild to moderate mitral regurgitation. Additionally, the echocardiogram detected the presence of a highly mobile filamentous structure, measuring 15 mm in length, located on the ventricular side of the aortic valve (as depicted in Figure 3B). This filamentous structure was determined to be the cause of moderate insufficiency in the aortic valve. No peripheral signs of IE, such as Janeway lesions or splinter hemorrhages, were identified.

**Figure 4:**
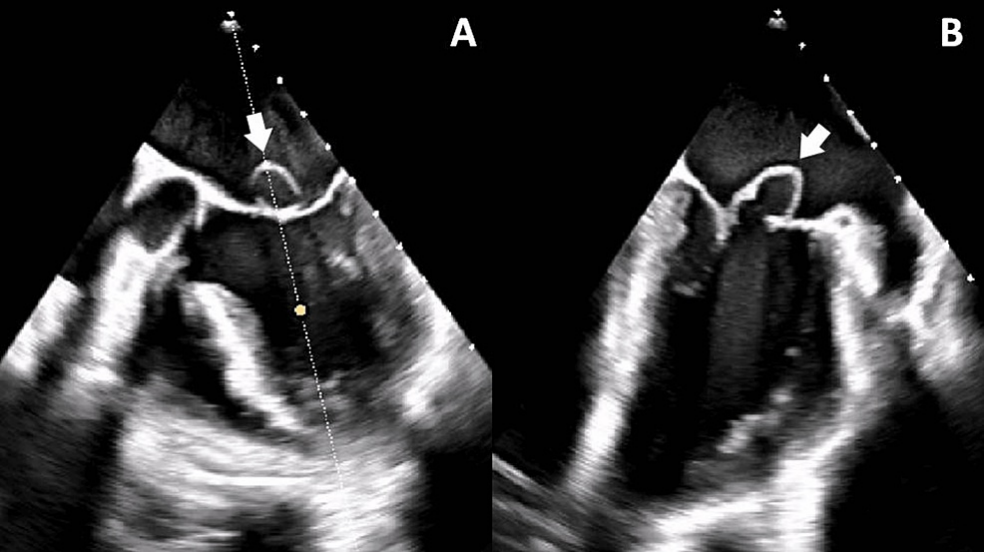
(A, B) Transesophageal echocardiogram revealing a pseudoaneurysm of the anterior mitral valve leaflet (arrows)

Upon receiving the diagnosis of mitroaortic endocarditis featuring large vegetations, a consultation was sought with cardiothoracic surgery. Their recommendation was that corrective valve surgery should be performed following six weeks of antibiotic therapy. To begin treatment, empirical antibiotics, including flucloxacillin, gentamicin, and ampicillin, were initiated. Subsequently, after the identification of *Streptococcus gallolyticus* in blood cultures, the antibiotic regimen was modified to penicillin and gentamicin. A favorable response to the treatment was observed, with the patient maintaining an afebrile status. In an effort to explore the possibility of bacterial translocation originating from an intestinal lesion, a colonoscopy was conducted, which revealed no abnormalities. Additionally, an orthopantomogram was performed, and the patient was evaluated by stomatology, ruling out any infectious focus or oral cavity lesions. Viral serological tests, including HIV, HBsAg, anti-HCV, and VDRL, yielded negative results. The patient’s electrocardiogram displayed sinus rhythm, carotid Doppler ultrasound did not reveal significant lesions in the neck vessels, and transcranial Doppler ultrasound indicated a residual 50% stenosis in the right M2 segment of the middle cerebral artery. Neurologically, an improvement in deficits was observed, with only residual right distal predominant brachial paresis characterized by a muscular strength of grade 3 in the hand and slight flattening of the right nasolabial fold. Follow-up cranioencephalic CT scans demonstrated resolving hematic densities without the presence of new lesions. For a more comprehensive characterization of the vegetation and valve changes, a transesophageal echocardiogram was performed. This examination identified vegetations on all three cusps of the aortic valve without the presence of abscesses, severe aortic insufficiency, and a pseudoaneurysm of the anterior mitral valve leaflet accompanied by associated vegetations. These findings ultimately resulted in mild to moderate mitral regurgitation due to leaflet deformation (as depicted in Figures 4A, 4B).

**Figure 5:**
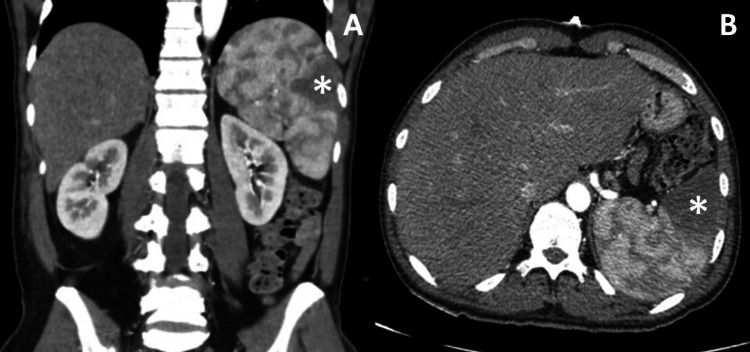
Abdominopelvic CT scan in the (A) coronal and (B) axial views showing extensive splenic infarctions (asterisks)

Recognizing the significant risk of pseudoaneurysm rupture, which could potentially lead to hemodynamic collapse, the case underwent reevaluation in consultation with cardiothoracic surgery. Taking into account the delicate balance between risks and benefits, the decision was reached to expedite the valve surgery intervention, scheduling it for two weeks following the hemorrhagic cerebral event. In order to monitor the abdominal findings, explore other areas susceptible to systemic embolization, and perform a thorough pre-valve surgery thoracic evaluation, a thoracoabdominopelvic CT scan was conducted. This imaging revealed a more extensive presence of splenic infarctions (as depicted in Figure 5) and identified images consistent with infarctions in the right kidney.

Given the exceedingly high risk of splenic hemorrhage during cardiac surgery, the approach underwent extensive discussion in a multidisciplinary meeting that included cardiothoracic surgery and general surgery. The consensus reached was to prioritize a splenectomy ahead of the valve intervention. Subsequently, a splenectomy was performed, but the post-operative period was marred by complications, specifically hemorrhagic shock. This critical situation necessitated an escalation in the level of care, prompting the patient’s transfer to the intensive care unit, with the need for invasive mechanical ventilation. The hemorrhagic focus was ultimately brought under control following a surgical revision involving the ligation of the splenic artery. In the days that followed, the patient’s condition deteriorated as they developed ventilator-associated pneumonia attributed to *Acinetobacter baumannii*. This infection progressed to septic shock and severe respiratory failure. Despite exhaustive efforts to provide multi-organ invasive support, including vasopressor and inotropic support, extracorporeal membrane oxygenation (ECMO), and renal replacement therapy, the relentless nature of the multisystemic disease eventually resulted in the unfortunate demise of the patient.

## Discussion

Systemic embolization in IE is a pivotal determinant of disease prognosis. Intracranial septic embolism resulting in large vessel ischemic stroke is the most frequent vascular cerebral complication [1]. The middle cerebral artery is the most common location for cerebral artery occlusion, accounting for over 90% of CNS emboli [3]. This manifestation is associated with a poor prognosis, especially in the absence of reperfusion [4]. In fact, mortality rates of IE-related strokes range from 20% to 60% in published series [1]. In what concerns the treatment of acute ischemic stroke in patients with IE, fibrinolysis carries a high risk of hemorrhagic complications and is not recommended [5]. However, it is sometimes performed in the absence of an IE diagnosis at the time of stroke onset. The application of mechanical thrombectomy (MT) in large vessel occlusion stroke related to IE is more contentious, with its safety and outcomes not being clearly established [4]. Despite the lack of strong evidence supporting its benefit in this particular setting, MT is practiced in selected cases and centers, with some case series presenting favorable results, suggesting it to be a safe procedure [2,4,6]. A retrospective multicenter study from five comprehensive stroke centers compared the outcomes of MT in stroke patients due to IE (28 patients) with MT in stroke patients presenting atrial fibrillation. In the case-control analysis, the authors concluded that the two groups presented similar safety and angiographic results, notably with no difference in terms of successful reperfusion (obtained in 85.7% of cases) or in terms of risk of symptomatic intracranial hemorrhage (occurring in 8% of cases) [4]. In this particular case, although thrombectomy led to the near-complete neurological recovery of the deficit, the patient presented with a minor hemorrhagic complication that is likely attributed to the fibrinolysis.

Characteristics associated with a higher risk of septic embolization to the CNS are, among others, the involvement of the anterior mitral valve leaflet, a vegetation exceeding 10 mm in size, and a significant mobility of the vegetation [1], all present in our patient. Besides the risk of new embolic events to the CNS or other territories, a very serious cardiac valvular involvement was present, with the potential to cause hemodynamic collapse. These characteristics indicate a necessity to consider valve intervention, although the decision regarding the optimal timing for surgical intervention is complex. In cases of left-sided native valve IE, there is a recommendation for early surgery in patients with destructive penetrating lesions, and it may also be considered for patients with mobile vegetations larger than 10 mm, particularly when involving the anterior mitral valve leaflet and when associated with other relative indications for surgery [1]. For patients who have already experienced a stroke, the decision for surgical intervention becomes even more intricate due to the risk of neurological deterioration caused by hemorrhagic transformation during cardiopulmonary bypass or exacerbation of cerebral ischemia due to intraoperative hypotension. In the case of cerebral hemorrhage, the risk is naturally heightened, making it reasonable to delay surgical intervention for up to four weeks after the event [1]. In this case, an earlier intervention was decided due to the severity of valvular complications.

In this case, despite the neurological symptoms and severe valve alterations at presentation, the unfavorable progression of the condition was ultimately linked to another complication, i.e., splenic embolization. The incidence of splenic involvement among patients with endocarditis is relatively common and includes hematoma, infarct, abscess, splenomegaly, and rarely splenic rupture [7]. Splenic rupture due to endocarditis is a life-threatening complication, especially after valve surgery, since anticoagulation used during cardiopulmonary bypass or following mechanic valve insertion may insidiously cause intrasplenic bleeding, hematoma enlargement, and eventually rupture [8]. Mortality rate in cases of splenic rupture following cardiac surgery for IE is reported to be around 30% in one study [7]. To prevent this, the conventional approach is splenectomy, and splenic artery embolization provides an effective and safe alternative, especially in the setting of a vulnerable cardiovascular status [7]. There are several reported cases of patients with IE and splenic abscesses in whom splenectomy was successfully performed in association with valve replacement (either four to five days after cardiac surgery or at the same operative time) [9]. Splenic artery embolization prior to life-saving valvular surgery was performed in a patient with IE complicated with heart failure and splenic hematoma, sparing the patient from a splenectomy [10]. This radiological intervention has also been successfully used to treat splenic rupture [7].

In what concerns the etiologic agent, it should be mentioned that *S. gallolyticus* (previously known as *S. bovis*) often originates from intestinal sources, necessitating colonoscopy to investigate the presence of malignancy or other mucosal lesions. In cases where *S. gallolyticus* is susceptible to penicillin, as in this case, the antibiotic response is highly favorable, with bacteriological cure rates ≥98% in patients completing four weeks of parenteral therapy [11]. This is in line with the clinical course of this patient, which became apyretic and presented decreasing acute phase reactants shortly after the implementation of antibiotic therapy.

## Conclusions

The intricacies of diagnosing and managing IE with embolic complications necessitate a multidisciplinary approach, involving various specialties such as internal medicine, infectious diseases, neurology, cardiothoracic surgery, and general surgery. Fibrinolysis is not recommended in IE-associated embolic stroke due to its inherent hemorrhagic risks, while thrombectomy appears to be effective and safe in cases of large vessel occlusion in this setting. Early cardiac surgery can be essential, especially when severe valvular dysfunction and high-risk embolic features are present. Decision-making for such intervention is intricate, particularly in patients with recent strokes, where both the risk of cerebral hemorrhage and the risk of worsening ischemia during cardiac surgery exist. In patients with significant splenic involvement, due to the risk of splenic rupture (either spontaneous or post-valvular intervention), a splenectomy or the embolization of the splenic artery should be considered.
